# Snow Control - An RCT protocol for a web-based self-help therapy to reduce cocaine consumption in problematic cocaine users

**DOI:** 10.1186/1471-244X-11-153

**Published:** 2011-09-25

**Authors:** Michael Schaub, Robin Sullivan, Lars Stark

**Affiliations:** 1 Research Institute for Public Health and Addiction, Zurich, Switzerland; 2 Working Group for the Low-Risk Use of Drugs, Zurich, Switzerland

## Abstract

**Background:**

Cocaine use has increased in most European countries, including Switzerland, and many states worldwide. The international literature has described treatment models that target the general population. In addition to supplying informative measures at the level of primary and secondary prevention, the literature also offers web-based self-help tools for problematic substance users, which is in line with tertiary prevention. Such programs, however, have been primarily tested on individuals with problematic alcohol and cannabis consumption, but not on cocaine-dependent individuals.

**Methods/Design:**

This paper presents the protocol of a randomised clinical trial to test the effectiveness of a web-based self-help therapy to reduce cocaine use in problematic cocaine users. The primary outcome is severity of cocaine dependence. Secondary outcome measures include cocaine craving, consumption of cocaine and other substances of abuse in the past month, and changes in depression characteristics. The therapy group will receive a 6-week self-help therapy to reduce cocaine consumption based on methods of Cognitive Behavioural Therapy, principles of Motivational Interviewing and self-control practices. The control group will be presented weekly psycho-educative information with a quiz. The predictive validity of participant characteristics on treatment retention and outcome will be explored.

**Discussion:**

To the best of our knowledge, this will be the first randomised clinical trial to test the effectiveness of online self-help therapy to reduce or abstain from cocaine use. It will also investigate predictors of outcome and retention. This trial is registered at Current Controlled Trials and is traceable as NTR-ISRCTN93702927.

## Background

Although data on the prevalence of problematic cocaine use and addiction are lacking in Switzerland and many other developed countries, there is no doubt that, in line with other countries, cocaine use has increased in Switzerland in recent years [1,2]. Over the past ten years, the number of cocaine-related disorder treatments has quintupled in outpatient treatment and advisory services [2]. In 2005, resident institutions reported that, for the first time in history, cocaine outstripped opiates as the main substance used [3]. This trend has also been observed in outpatient units [3]. Further evidence of increased cocaine consumption has been found by quantifying cocaine concentrations in sewage effluents [4] and in recent HBSC student surveys [5]. The abovementioned increase in treatment requests likely reflects only a minority of cocaine users. Presumably, the majority of users consume cocaine on a quasi-controlled basis, whereas only a small fraction of consumers is likely to take advantage of available treatments [6]. However, it is expected that some users will switch from controlled to problematic use [7]. For those users, interventions that follow the principle of concurrent cover (i.e., non-invasive, low-cost interventions in which therapeutic intensity can be enhanced according to need) appear appropriate.

In recent times, the international literature has described treatment models that target the general population. In addition to supplying informative measures at the level of primary and secondary prevention, the literature also offers web-based self-help tools for problematic substance users, which is in line with tertiary prevention [8-10].

Web-based self-help programs that reduce problematic consumption are able to reach "hidden" consumer groups in the general population due to their low treatment threshold and non-restrictive setting for intervention [11]. Furthermore, these programs show a remarkably positive cost-benefit relation [12], which is of interest in Switzerland and other industrialised countries suffering from exorbitant health costs. Such programs, however, have been primarily tested on individuals with problematic alcohol and cannabis consumption but not on cocaine-dependent individuals [9,10,13].

Therefore, Snow Control, a six-week self-help therapy for problematic cocaine users who intend to reduce or stop consuming and have access to the Internet was developed in 2010. Snow Control is based on methods of Cognitive Behavioural Therapy (CBT) that have been tested on cocaine addicts [14,15], principles of Motivational Interviewing [16], current self-control practices and the established Relapse-Prevention Model [17-19].

The therapy is structured into three parts and includes the following eight modules that are activated for access week by week (modules 1 to 4) and four additional voluntary modules (module 5 to 8) that can be activated during week 4-6:

• Part 1: Introduction

○ Registration process

○ Explanation of the cocaine consumption diary and its fully automated progress charts and statistics

○ Examination of the pros and cons resulting from a change in cocaine consumption patterns to address motivation

○ Explanation of the "My Snow Control" folder (This folder allows individuals to review the acquired summarised module documents, e.g., the list of the top five strategies for dealing with cocaine cravings)

• Part 2: Key Modules (to be worked through in the following order)

○ Module 1: Strategies for goal achievement

○ Module 2: Identifying risk situations

○ Module 3: Dealing with cocaine craving

○ Module 4: Dealing with relapses

• Part 3: Further Modules (to be worked through in optional order but with the recommendation to complete at least two)

○ Module 5: Enjoying leisure time

○ Module 6: Dealing with burdens

○ Module 7: Saying "no" to foster refusal skills

○ Module 8: Preserving achievements

After the completion of part 1, each login in the therapy group will direct the participant to the consumption diary. The participants are asked to determine the amount of cocaine they plan to consume in the next 7 days and to specify the amount of cocaine consumed in the past 7 days into their consumption diary. After the completion of the consumption diary, they are directed to their weekly module (part 2 to be worked through in the above mentioned order; part 3 to be worked through in an optional order).

To assess the effectiveness of the Snow Control therapy, an appropriate psycho-educative online control condition was developed. Participants in the control condition receive eight psycho-educative information modules on the risks, potential harms and other important information about cocaine consumption. The frequency of the control condition is comparable to the 6 weeks of intervention; however, it does not include the presentation of a consumption diary first. After having read each of the information modules, the participants are invited to participate in a weekly quiz to evaluate their information knowledge.

## Methods/Design

### Aims of the trial

This study aims to test the effectiveness of the web-based cognitive-behavioural self-help therapy Snow Control to reduce cocaine use in problematic cocaine users in a two-arm randomised controlled trial. The primary outcome is the change in severity of cocaine dependence between baseline, 3 and 6 weeks of therapy/intervention and at a 6-month follow up. The secondary outcome measures include changes in cocaine craving, the past month's consumption of cocaine and other substances of abuse and changes in depression characteristics. The predictive validity of participant characteristics for treatment retention and outcome will be explored.

### Study population

The study population will be recruited through the Snow Control website, several websites from local outpatient treatment centres and from nightlife prevention websites. In addition, advertisements in Internet-forums and newspapers will be traced.

### Hypotheses

We hypothesise that Snow Control participants in the therapy group will show higher reductions on the Severity of Dependence Scale (SDS) [20] sum score and cocaine consumption than those in the control group at the 6-week treatment termination and at the 6-month follow up. Moreover, we expect participants in the therapy group to improve more significantly with respect to the secondary outcomes between baseline and 6-week treatment termination. We also expect the participants in the therapy group to show significantly higher retention.

### Measurement instruments

The primary outcome instrument, the Severity of Dependence Scale (SDS), is a 5-item questionnaire that indicates the severity of dependence on cocaine. Each of the five items is scored on a 4-point scale (0-3). The total score is obtained through the addition of the 5 item ratings. High scores indicate a high level of dependency.

Moreover, the following secondary outcome instruments will be applied: 1) The Cocaine Craving Questionnaire Brief CCQ-B [21] is a short (10 items) and validated instrument that was derived from the CCQ-Now [22] (45 items). It contains 10 craving symptoms that are rated on a 7-point Likert scale from strongly agree to strongly disagree. 2) The "Fragebogen Substanzanamnese" (FDA) ascertains the years of lifetime consumption, the past month's consumption, and the way of consumption for the DSM-IV/ICD-10 substances of abuse. This measure was derived from the EuropeASI [23]. 3) The short version of the Beck Depression Inventory (BDI-V) [24] is a derived, validated, and user-friendly short version of the classical Beck Depression Inventory. The BDI-V contains 20 items with a 6-point Likert scale (0/never-1-2-3-4-5/almost every time). Accordingly, the values range from 0 to 100, with a cut-off of 45 for a serious depressive episode that requires further treatment.

### Estimation of the Expected Effect Sizes and Sample Size

The maximal SDS score is 15 points, and the average SDS standard deviation in previous studies was 5 points. As we expect relatively large cocaine consumption differences between participants, we expect a conservative SDS deviation of 7.5 points. For a successful reduction in cocaine use, an average 25% SDS score reduction (3.75 points reduction for a small to medium effect size) is anticipated. This results in a total sample of 25 participants in each group (α = .05, 1-β = 0.8). According to the pilot study, we expect 70% of the participants to quit the study before completion at six weeks. We thus aim to recruit a total of 170 participants at baseline.

### Consent Procedure

The rationale of the study will be explained to the participants. They will also be informed about the different assessments, assessment schedules and duration. The participants will then be informed about (1) study inclusion and exclusion criteria (see table 1), (2) the potential risks of participation, (3) safety arrangements during and after the study phase, (4) the inability of Snow Control to replace face-to-face therapy for problematic cocaine use/abuse, and (5) the circumstances under which they should contact their general practitioner or a professional from the medical advisory and emergency list that will be accessible at all times and how to make this contact. The participants will also be informed that the study has been reviewed by the ethic committee of the Canton of Zurich and given their declaration of no objection (nihil obstat). Moreover, they will be informed about their right to withdraw from the study at any time without consequences except for the loss of further compensation. Informed consent will be accepted when participants click on a field on the informed consent page and submit the consent with a submission button.

**Table 1:** Inclusion and exclusion criteria and reasoning

*Inclusion Criteria*	Reasoning
- Minimal age of 18 years	To ensure a minimal age of participation
- Cocaine use > 2 occasions in the last 30 days	To include occasional users in order to provide extended study validity
***Exclusion Criteria***	**Reasoning**
- Participation in other psycho-social or pharmacological treatments for the reduction/cessation of cocaine use	To avoid confounding of treatment effects
- Opioid use in the last 30 days (exception: substitution maintenance treatment for opioid dependence without heroin use in the last 30 days)	To avoid confounding of drug effects
- Ever been in treatment for cardiovascular problems or apoplexy	To avoid subjects with these problems entering the study
- BDI-V score > 45	To avoid subjects with serious symptoms of depression entering the study

### Baseline Assessment

After providing informed consent, subjects who meet study entry criteria will create a personal and secure login and password (with automated real-time verification of the passwords' security level) and will receive an automated e-mail notification with their access information. They will then be directed to the baseline assessment on socio-demographic characteristics and consumption patterns (see table 2). Participants that do not meet the inclusion and/or meet one of the exclusion criteria (see table 1) will receive an explanation about why they are not permitted to participate in the study and be provided recommendations (e.g., not to reduce their consumption of cocaine before visiting a physician to receive more accurate treatment, etc.). A corresponding decision tree for the possible inclusion and exclusion criteria combinations will be constructed and implemented. The completion of the baseline assessment will allow participants to begin the Snow Control therapy or the control tool according to an automated online allocation procedure. Participants that do not fulfil the criteria can proceed with the Snow Control therapy modules, though without study participation (no assessments and no compensation).

**Table 2:** Measurements and instruments

Assessments/instruments	Baseline	3 weeks	6 weeks	6-month follow up
Socio-demographics	x			
Previous psychiatric and somatic treatments	x			
SDS	x	x	x	x
CCQ-B	x	x	x	x
FDA	x	x	x	x
BDI-V	x		x	x

### Randomisation and Allocation

Once participants have completed their baseline assessment, they will be randomly brought to part 1 of either the intervention or the control tool, and this assignment will be automatically registered in the background database. This assignment will also be registered in their Internet browser as a cookie to avoid multiple registrations by one person. If a person returns to the Snow Control start page and attempts to register for a second time, she or he will be recognised by the background database and automatically be redirected to his or her allocation.

### Safety

During the 6-week therapy/intervention phase, participants will have the opportunity to contact a corresponding outpatient clinic in a nearby city by telephone (lists with opening hours, web-links, postal addresses, and telephone numbers will constantly be provided in the corresponding language). In addition, a medical advisory and emergency list in case of an emergency will be provided according to the web-based treatment guidelines from the Federation of Swiss Psychologists [25] (in line with the HONcode [26], a code of ethics for medical information on the Internet). This list will always be accessible before, during, and after (pdf-version for print out) the study participation regardless of whether they withdraw or drop out of the study. This list will include numbers of emergency help lines and the contact information of the study team and the webmaster.

### Trial Flow

**Figure 1:**
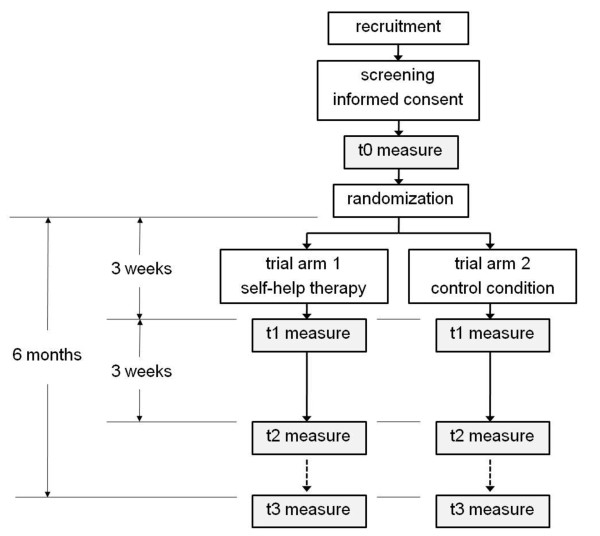
**Trial flowchart. This figure provides an overview of the participant flow for this trial**.

Figure 1 provides an overview of the trial flow. If a participant successfully completes the baseline assessment (t0), he or she will be introduced step-by-step into either the intervention or the control tool (part 1) and invited to participate in module 1. Every week and two days in advance to the next module, a participant will receive an automated e-mail notification to login and return to the next module. Modules 1 to 4 will be accessible only week by week. After three weeks, the automated e-mail will direct the participant to the intermediate assessment (t1) before he or she has access to module 4 of the intervention or the control tool (end of part 2). Six weeks after the baseline assessment, completion of the additional optional modules of part 3 and weekly reminder e-mail notifications, participants will be invited again by e-mail to login and complete the final study assessment (t2). It is possible that some participants will wish to continue with some of the modules in part 3 after this period. However, the decision has been made to have the follow up timing depend solely on the time interval since t0. In our opinion, this is the best possible adherence to the 'intention to treat' principle. For the follow up assessment (t3), participants will be invited by an e-mail message 6 months past t0, with the notification that completion of the entire 6-month follow up assessment will be compensated by a 40 Euro incentive (an online voucher or an online charitable donation).

### Handling of study dropouts

Each week, participants will be sent an automated e-mail that contains a reminder to work on the next module and a direct link to the Snow Control login site. If participants do not log in, they will receive a reminder e-mail every three days within the following two weeks. If they do not continue their participation after these reminders, they will be considered to have dropped out of the study. Participants who skip one of the study questionnaires by not answering all of the questions will be identified in the data analyses and counted as dropout (cut-off: answered at least 70% of the questions).

### Data Analysis

Data will be analysed according to the intention-to-treat principle. Multiple imputations of missing data handling procedures will be implemented using the statistical software package STATA (version 10). Baseline measurements will be compared using t- and Chi-squared tests. Differences between primary and secondary outcome variables at three and six weeks will be tested using a repeated measures ANOVA. Effect sizes will be calculated using Cohen's d [27]. Differences in treatment retention will be tested using multiple logistic regression analyses. We will additionally conduct explorative regression analyses in order to test whether baseline variables predict cocaine abstinence, cocaine craving (CCQ-B), or reduced symptoms of depression (BDI-V). For these analyses, we will use linear, multinomial, or binary regression models dependent on the scale level of the outcome measures.

### Ethical Review

This RCT will be executed in compliance to the Helsinki Declaration and has been reviewed by the ethic committee of the Canton of Zurich and given their declaration of no objection (KEK-StV-Nr.70/09).

## Discussion

To the best of our knowledge, this is the first randomised controlled trial to test the effectiveness of a web-based self-help therapy to reduce or abstain from cocaine use in problematic cocaine users. It will also be the first study to explore the predictors of outcome and retention in this type of therapy for problematic cocaine users. If the efficacy of this therapy is demonstrated in this RCT, Snow Control will be integrated into the basic services for cocaine users, and we will potentially be able to reach "hidden" cocaine consumer groups in the general population [11] in a remarkably positive cost-benefit relation [12].

A specific problem includes the online implementation of informed consent. We will rely on participants to click on a field on the informed consent page and submit the consent with a submission button. We must thus trust that participants have read and understood the study information and that they are at least 18 years old. However, as we followed the considerations of [28] and [29] on how to best implement the informed consent procedure online, we expect the participants to give their full attention to reading the study information and providing informed consent. Nevertheless, the problem of minors under the age of 18 participating in the study cannot be addressed further.

A further point of concern regarding the accuracy of the participants' online responses is the self-reported nature of online substance use information. A number of studies have shown that self-reported information regarding substance use, especially cocaine use, is reliable [30-32]. As participants provide substance use information within a secure password-protected online environment and presumably in a secure physical environment, such as at home in front of their own computers, we expect reliable information.

Another point of concern is that the intended multivariate statistics rely considerably on a normal data distribution, and the data may be skewed as we defined frequencies of cocaine consumption in the inclusion criteria. Therefore, multivariate statistics should be performed with caution. As suggested by our Dutch colleagues [8] in their RCT protocol for the evaluation of real-time internet therapy vs. online self-help for problematic alcohol users, we will utilise bootstrap methods and permutation tests according to Hesterberg and colleagues [33].

During the online implementation of the consumption diary of the Snow Control therapy group, we considered asking pilot participants to note the number of cocaine lines consumed. The pilot tests showed that although this includes some numeracy skills, pilot participants did not feel disturbed to note their consumption in grams of cocaine consumed per day. However, because the well-established SDS is utilised as the main outcome and the CCQ-B as a secondary instrument with a randomised control group, we do not expect much influence of purity and quality of the cocaine consumed or the methods of consumption to interfere with the potential outcome differences.

Finally, a potential problem for this trial is the expected high number of lost participants at the end of and at follow up of the online therapy, which will likely be even higher in the control intervention. We will address this issue in three ways. 1) All participants must invest approximately 30 minutes for the baseline assessment, which will select rather motivated participants and prevent the participation of un-motivated participants. 2) Participation at 6 months follow up assessment will be compensated by a 40 Euro incentive (an online voucher or an online charitable donation). 3) All missing values in the final data set will be multiple-imputed, a promising approach that, as shown by our Dutch colleagues, has become increasingly important in e-health research [34].

## Competing interests

The authors declare that they have no competing interests. This trial is registered at Current Controlled Trials and traceable as NTR-ISRCTN93702927.

## Authors' contributions

MS was responsible for the study design, prepared the first draft of the paper and the final manuscript. MS, RS and LS developed the Snow Control self-help therapy and the control condition. RS programmed and implemented the study website. All of the authors approved the final version of the manuscript submitted for publication.

## Pre-publication history

The pre-publication history for this paper can be accessed here:

http://www.biomedcentral.com/1471-244X/11/153/prepub

## References

[B1] Bruggisser M Ceschi A Bodmer M Wilks MF Kupferschmidt H Liechti ME Retrospective analysis of stimulant abuse cases reported to the Swiss Toxicological Information Centre during 1997-2009 Swiss Med Wkly 2010 140 w13115 21188679 10.4414/smw.2010.13115

[B2] Maag V Kokain - die neue Volksdroge? Nationale und internationale Trends Abhängigkeiten 2006 13 2221926583

[B3] Act-info Act-info Jahresbericht 2010 - Suchtberatung und Suchtbehandlung in der Schweiz. Ergebnisse des Monitoringsystems 2011 Bern: Bundesamt für Gesundheit

[B4] UNODC World Drug Report 2007 United Nations, Office on Drug Crime

[B5] Schmid H Delgrande-Jordan M Der Konsum psychoaktiver Substanzen von Schülerinnen und Schülern in der Schweiz - Ausgewählte Ergebnisse einer Studie, durchgeführt unter der Schirmherrschaft der Weltgesundheitsorganisation (WHO) (Forschungsbericht Nr. 42) 2007 Lausanne: Schweizerische Fachstelle für Alkohol- und andere Drogenprobleme (SFA)

[B6] Prinzleve M Haasen C Zurhold H Matali JL Bruguera E Gerevich J Bácskai E Ryder N Butler S Manning V Gossop M Am Pezous Verster A Camposeragna A Andersson P Olsson B Primorac A Fischer G Güttinger F Rehm J Krausz M Cocaine use in Europe - a multi-centre study: patterns of use in different groups Eur Addict Res 2004 10 147 155 10.1159/000079835 15367815

[B7] Haasen C Prinzleve M Zurhold H Rehm J Güttinger F Fischer G Jagsch R Olsson B Ekendahl M Verster A Camposeragna A Pezous A Gossop M Manning V Cox G Ryder N Gerevich J Bacskai E Casas M Matali JL Krausz M Cocaine use in Europe - a multi-centre study. Methodology and prevalence estimates Eur Addict Res 2004 10 139 146 10.1159/000079834 15367814

[B8] Blankers M Koeter M Schippers GM Evaluating real-time internet therapy and online self-help for problematic alcohol consumers: a three-arm RCT protocol BMC Public Health 2009 9 16 10.1186/1471-2458-9-16 19144162 PMC2636801

[B9] Postel MG de Haan HA ter HED Becker ES de Jong CA Effectiveness of a web-based intervention for problem drinkers and reasons for dropout: randomized controlled trial J Med Internet Res 2010 12 e68 21163776 10.2196/jmir.1642 PMC3056532

[B10] Riper H Kramer J Smit F Conijn B Schippers G Cuijpers P Web-based self-help for problem drinkers: a pragmatic randomized trial Addiction 2008 103 218 227 10.1111/j.1360-0443.2007.02063.x 18199300

[B11] Cunningham JA Humphreys K Koski-Jannes A Cordingley J Internet and paper self-help materials for problem drinking: is there an additive effect? Addict Behav 2005 30 1517 1523 10.1016/j.addbeh.2005.03.003 15893433

[B12] Curry SJ eHealth research and healthcare delivery beyond intervention effectiveness Am J Prev Med 2007 32 S127 S130 10.1016/j.amepre.2007.01.026 17466817

[B13] Copeland J Martin G Web-based interventions for substance use disorders: a qualitative review J Subst Abuse Treat 2004 26 109 116 10.1016/S0740-5472(03)00165-X 15050088

[B14] Carroll KM Rounsaville BJ Nich C Gordon LT Wirtz PW Gawin F One-year follow-up of psychotherapy and pharmacotherapy for cocaine dependence. Delayed emergence of psychotherapy effects Arch Gen Psychiatry 1994 51 989 97 7979888 10.1001/archpsyc.1994.03950120061010

[B15] Carroll KM Recent advances in the psychotherapy of addictive disorders Curr Psychiatry Rep 2005 7 329 336 10.1007/s11920-005-0032-5 16216150

[B16] McKee SA Carroll KM Sinha R Robinson JE Nich C Cavallo D O'Malley S Enhancing brief cognitive-behavioral therapy with motivational enhancement techniques in cocaine users Drug Alcohol Depend 2007 91 97 101 10.1016/j.drugalcdep.2007.05.006 17573205 PMC2386854

[B17] Sobell MB Sobell LC Problem drinkers: Guided self-change treatment 1993 New York: Guilford Press

[B18] Sanchez-Craig M Saying When: How to Quit Drinking or Cut Down 1993 Toronto: Addiction Research Foundation

[B19] Velicer WF Diclemente CC Rossi JS Prochaska JO Relapse situations and self-efficacy: an integrative model Addict Behav 1990 15 271 83 10.1016/0306-4603(90)90070-E 2378287

[B20] Gossop M Darke S Griffiths P Hando J Powis B Hall W Strang J The Severity of Dependence Scale (SDS): psychometric properties of the SDS in English and Australian samples of heroin, cocaine and amphetamine users Addiction 1995 90 607 614 10.1111/j.1360-0443.1995.tb02199.x 7795497

[B21] Sussner BD Smelson DA Rodrigues S Kline A Losonczy M Ziedonis D The validity and reliability of a brief measure of cocaine craving Drug Alcohol Depend 2006 83 233 237 10.1016/j.drugalcdep.2005.11.022 16384655

[B22] Tiffany ST Singleton E Haertzen CA Henningfield JE The development of a cocaine craving questionnaire Drug Alcohol Depend 1993 34 19 28 10.1016/0376-8716(93)90042-O 8174499

[B23] Kokkevi A Hartgers C Europe ASI: European adaptation of a multidimensional assessment instrument for drug and alcohol dependence Eur Addict Res 1995 1 208 210 10.1159/000259089

[B24] Schmitt M Altstötter-Gleich C Hinz A Maes J Brähler E Normwerte für das Vereinfachte Beck-Depressions-Inventar (BDI-V) in der Allgemeinbevölkerung Diagnostica 2006 52 51 59 10.1026/0012-1924.52.2.51

[B25] Föderation der Schweizer Psychologinnen und Psychologen FSP Qualitätskriterien für psychologische Angebote im Internet http://www.psychologie.ch/fileadmin/user_upload/dokumente/berufspolitik/d-qual-krit-inet-06.pdf

[B26] Health on the Net Foundation HON The HON Code of Conduct for medical and health Web sites (HONcode) http://www.hon.ch/HONcode/Conduct.html

[B27] Cohen J Statistical power analysis for the behavioral sciences 1988 2 Hillsdale, NJ: L. Erlbaum Associates

[B28] Keller HE Lee S Ethical issues surrounding human participants research using the Internet Ethics Behav 2003 13 211 219 10.1207/S15327019EB1303_01 14680001

[B29] Varnhagen CK Gushta M Daniels J Peters TC Parmar N Law D Hirsch R Sadler Takach B Johnson T How informed is online informed consent? Ethics Behav 2005 15 37 48 10.1207/s15327019eb1501_3 16127857

[B30] Vitale SG van de Mheen H van de Wiel A Garretsen HF Substance use among emergency room patients: Is self-report preferable to biochemical markers? Addict Behav 2006 31 1661 1669 10.1016/j.addbeh.2005.12.011 16446045

[B31] Calhoun PS Sampson WS Bosworth HB Feldman ME Kirby AC Hertzberg MA Wampler TP Tate-Williams F Moore SD Beckham JC Drug use and validity of substance use self-reports in veterans seeking help for posttraumatic stress disorder J Consult Clin Psychol 2000 68 923 927 11068979

[B32] Zaldivar BF Garcia MJM Flores CP Sanchez SF Lopez RF Molina MA Validity of the self-report on drug use by university students: correspondence between self-reported use and use detected in urine Psicothema 2009 21 213 219 19403073

[B33] Hesterberg T Moore DS Monaghan S Clipson A Epstein R Moore DS, MacCabe GP Bootstrap Methods and Permutation Tests Introduction to the Practice of Statistics 2005 14 5 New York: WH Freeman & Company 1 70

[B34] Blankers M Koeter MW Schippers GM Missing data approaches in eHealth research: simulation study and a tutorial for nonmathematically inclined researchers J Med Internet Res 2010 12 e54 21169167 10.2196/jmir.1448 PMC3057309

